# Model Construction and Prediction of Combined Toxicity of Arsenic(V) and Lead(II) on *Chlamydomonas reinhardtii*

**DOI:** 10.3390/biology14101395

**Published:** 2025-10-11

**Authors:** Zhongquan Jiang, Tianyi Wei, Chunhua Zhang, Xiaosheng Shen, Zhemin Shen, Tao Yuan, Ying Ge

**Affiliations:** 1 East China Sea Fisheries Research Institute, Chinese Academy of Fishery Sciences, Shanghai 200090, China; zhongquanjiang@outlook.com; 2 College of Resources and Environmental Sciences, Nanjing Agricultural University, Nanjing 210095, China; 3 State Environmental Protection Key Laboratory of Environmental Health Impact Assessment of Emerging Contaminants, School of Environmental Science and Engineering, Shanghai Jiao Tong University, Shanghai 200240, China; sardine0722@sjtu.edu.cn (T.W.); zmshen@sjtuedu.cn (Z.S.); taoyuan@sjtu.edu.cn (T.Y.); 4 Laboratory Centre of Life Science, College of Life Science, Nanjing Agricultural University, Nanjing 210095, China; chunhua@njau.edu.cn

**Keywords:** arsenic(V), lead(II), model prediction, combined toxicity, *Chlamydomonas reinhardtii*

## Abstract

**Simple Summary:**

Arsenic and lead, toxic metal and metalloid from industry and agriculture, often pollute water together and harm aquatic life, but we do not fully understand how they act together. We looked at how these metals affect the growth of a common algae called *Chlamydomonas reinhardtii* under laboratory conditions and used models to predict their combined harm. We found arsenic(V) is much more toxic than lead(II), and as arsenic levels rise in mixtures, the two metals go from acting together to being more harmful than expected. Under the environmentally relevant ratio of As:Pb = 1:10, the mix badly damages algae cells. These findings help us better assess water pollution risks and create better strategies to protect aquatic ecosystems and human health.

**Abstract:**

With the acceleration of industrialization, the impact of the toxic metalloid arsenic (As) and metal lead (Pb) on aquatic ecosystems has garnered widespread concern. However, the specific toxic effects of how these two metals jointly impact aquatic organisms are not yet fully understood. This study aims to investigate the toxic effects of As and Pb individually and in combination of the mixture on the growth of *Chlamydomonas reinhardtii* (*C. reinhardtii*) in a lab setup using the Concentration Addition (CA) model and the Independent Action (IA) model to predict the toxic effects at different concentrations. The results indicated that As and Pb had significant inhibitory effects on the growth of algae, and the toxicity of As was greater than that of Pb (As EC50 = 374.87 μg/L, Pb EC50 = 19,988.75 μg/L), measured by Spectrophotometer. As the metal concentrations increased, both metals demonstrated classic sigmoidal concentration-effect curves. Furthermore, we discovered that in mixtures of As and Pb at varying concentration ratios, the combined toxic effect shifted from additive to synergistic with increasing As concentration, exhibiting a pronounced concentration ratio dependency. Utilizing nonlinear least squares regression, we successfully constructed concentration-response models for both As and Pb, employing Observation-based Confidence Intervals (OCIs) to reflect the uncertainty of the data. By comparing experimental data with model predictions, the EC50 was used as an index to compare the toxicity magnitude of As/Pb mixtures. The toxicity of As and Pb mixtures gradually increases with the increase in their concentration ratios. Scanning and transmission electron microscopic observations revealed that the combination of 200 μg/L As and 2000 μg/L Pb resulted in the greatest synergistic toxic effect, with severe breakage and indentation to *C. reinhardtii* cells. This study not only provided new insights into the environmental behavior and ecological risks of As and Pb but also held significant implications for effective water pollution management strategies by offering a validated model-based framework for predicting mixture toxicity across different concentration regimes.

## 1. Introduction

The proliferation of industrial activities worldwide has led to a significant increase in heavy metal and metalloids pollution, posing a profound threat to environmental integrity and public health [1,2,3]. These elements are commonly found in industrial effluents, agricultural runoff, and urban wastewater, and through surface runoff, they infiltrate aquatic ecosystems, presenting a clear and present danger to the health of these environments [2]. Among the various heavy metals, arsenic (As) and lead (Pb) are particularly concerning due to their prevalence and toxicity [4]. Arsenic and lead are frequently found together in polluted environments due to shared sources such as mining activities, industrial discharge, and the use of arsenic- and lead-containing pesticides. These shared sources result in combined contamination in natural waters, which can exacerbate toxic effects on aquatic organisms [5,6]. Aquatic organisms, encompassing microorganisms, lower plants, and animals, are directly impacted by the toxic effects of arsenic (As) and lead (Pb), which can disrupt cellular processes, inhibit growth, and result in mortality [4,7,8]; moreover, these heavy metals pose a direct threat to the integrity of aquatic ecosystems and, through bioaccumulation and biomagnification in the food chain, may ultimately endanger human health [3,9,10].

Moreover, the heavy metals different mixture components in water may pose a higher toxicity risk to aquatic organisms, the presence of multiple metals may lead to additive, synergistic, antagonistic and independent effects, so the combined toxicity between environmental pollutants has gradually become a hot topic of environmental and health concerns [9,11,12]. As-Cu co-exposure has a synergistic effect on the toxicity of microalgae, mainly because Cu promotes the absorption of As by algae [13]. The study indicates that the toxic response of Microcystis aeruginosa to Cd and Pb is not a simple antagonistic effect, but rather a differential sensitivity dependent on the type and concentration of the metals. Low concentrations (1–5 mg/L) of Cd and Pb can gradually enhance chlorophyll fluorescence, while higher concentrations (20 mg/L Cd and 10–20 mg/L Pb) lead to a significant decrease in chlorophyll levels and fluorescence [14]. Combined toxicity can interfere with multiple cellular processes of algae, including changing the spatial structure of membrane proteins and regulating membrane fluidity, thereby affecting photosynthesis and energy transfer, as well as major metabolic disorders, causing a decrease in the algae’s electron transfer rate and cell density, photosynthesis rate decreases and chlorophyll content significantly decreases [8,15]. The proportion of pollutants in the natural environment is numerous and complex, so the selection of heavy metal concentration ratio is the first key point in studying the toxicity of environmental pollutants [16]. The studies have shown that the co-existence of multiple heavy metals (in various dissolved forms/compounds) can produce complex interactions, resulting in additive, synergistic, or even antagonistic effects on toxicity. For instance, arsenic and copper co-exposure has been shown to intensify toxicity in microalgae due to copper’s role in promoting arsenic uptake [13]. However, research specifically addressing the joint effects of arsenic and lead is limited, and existing studies often lack predictive models capable of accurately forecasting toxicity under varying concentration ratios. This gap highlights the need for a systematic investigation into the combined toxicity of arsenic and lead to improve environmental risk assessment models [17].

*Chlamydomonas reinhardtii* (*C. reinhardtii*), as a model organism, is an ideal model alga for use in the study of heavy metal toxicity due to its rapid growth, ease of cultivation, and genetic manipulability [18,19]. *C. reinhardtii* has been shown to have some degree of resistance to the toxicity of single forms of arsenic (As(V)) and lead (Pb(II)), with EC_50_ values of 75 mg/L for As(V) and 1.6 g/L for Pb(II) [20,21]. In addition, the combined toxicity of different levels of As(V) and Pb(II) to *C. reinhardtii* has also been investigated at the physiological level. The previous study of our group found that under the coexistence condition of high levels of As(V) and Pb(II) (500 µg/Las(V) + 5000 µg/L Pb(II)), the presence of Pb(II) significantly inhibited the exocytosis of As, and this interaction triggered a synergistic toxicity effect, which resulted in the cells of the alga *C. reinhardtii* experiencing severe physiological stress, which may ultimately lead to destruction of cellular structures and cell lysis [8,15]. Although studies have reported single and combined toxic effects of each arsenic and lead, studies on the combined effects of these two metallic elements are relatively limited, especially in predicting toxic effects at different concentrations.

The aim of this study was to predict and evaluate the combined toxicity of As(V) and Pb(II) treatments on *C. reinhardtii*. The cells are used in the production of pharmaceutical proteins, the preparation of bioenergy, and the physiological mechanisms of response to heavy metal stress. To identify and quantify the nature of the toxic interaction between As and Pb, the growth inhibition rate of *C. reinhardtii* at different mixed concentration ratios of As and Pb was determined under 96 h conditions. Combined with the ratio of binary mixtures of As and Pb in the actual environment, a representative As:Pb = 1:10 concentration ratio was selected for toxic effect and model prediction analysis, and the observed toxicity was compared with the toxicity predicted by CA and IA models to evaluate their mixtures. Moreover, the morphology of the *C. reinhardtii* cells was observed by scanning electron microscope (SEM) and transmission electron microscope (TEM). This provides a reference for the development of effective water pollution management strategies and preventive measures to mitigate the impact of heavy metal pollution on aquatic ecosystems and human populations.

## 2. Materials and Methods

### 2.1. Algal Strain and Culture Conditions

*C. reinhardtii* (CC-125) was purchased from the Chlamydomonas Resource Center of the Department of Plant and Microbial Biology, University of Minnesota (https://www.chlamycollection.org/, accessed on 31 February 2024). The algae were cultured in TAP medium (Tris-Acetate-Phosphate medium), whose composition is detailed in Supplementary Table S1. The growth conditions were as follows: light/dark 12 h:12 h, temperature 25 °C, rotational speed 120 rpm and light intensity 2000 Lux. They were continuously cultured in constant temperature and light oscillation incubator for 96 h based on preliminary experiments (see Figure S1A). Culture stability was maintained by using sterile medium, periodically checking for contamination via microscopy, and using fresh cultures in the logarithmic growth phase for all experiments. Then, each sample (200 μL) was analyzed for its OD_680_ using the Molescular Devices (SpectraMax i3X, San Jose, CA, USA), which corresponds to the maximum light absorption peak of chlorophyll *a* (see Figure S1B).

### 2.2. As and Pb Treatments

A 1000 mg/L As(V) stock solution (o2si smart solutions) was diluted to prepare the As(V) for treatments. The Pb (NO_3_)_2_ powder (analytical grade, Xilong Scientific, Shantou, China) was used to make 10,000 mg/L Pb(II) stock solution, which was diluted to prepare the Pb(II) treatment solutions.

According to the preliminary toxicity range-finding tests, the maximum effect concentration (*C_H_*, growth inhibition effect E ≥ 98%) and the minimum effect concentration (*C_L_*, growth inhibition effect E ≤ 1%) of single or binary mixed ionic solution were determined.

The concentration-effect data of single or mixed ions were usually obtained by gradually diluting a high concentration solution into a series of low concentrations. For the pollutants with logarithmic linear relationship between concentration and effect, the concentration gradient with uniform distribution was obtained by dilution factor. According to the concentration-effect relationship of different pollutants, the dilution factor *F* was calculated by the following Equation (1).

(1) $$F{=(\frac{C_{L}}{C_{H}})}^{\frac{1}{n-1}}$$

So, the concentration (*Cn*) of the 12 gradient points is:

(2) $$C_{n}=C_{1}\times F^{n-1}\;\;\;n=1,\;2\cdots \cdots 12$$

where *C_1_* = *C_H_* (Maximum effect concentration), *C_12_* = *C_L_* (Minimum effect concentration).

**Figure 1:**
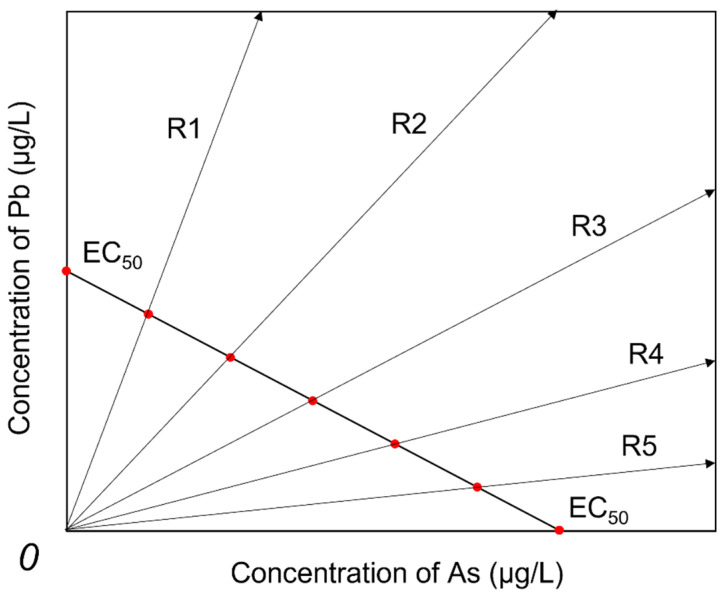
Schematic diagram of direct equipartition ray design, the red dots in the figure mark the equal division points of the line segments.

This study adopts a robust method for studying the concentration-effect of binary mixtures, the direct equipartition ray design (EquRay) method [16]. Five mixture ray concentration ratios were designed for the binary mixture of As(V) and Pb(II) according to EquRay (see Figure 1). The concentration ratios of the five rays are 5EC_50,As_:EC_50,Pb_, 4EC_50,As_:2EC_50,Pb_, 3EC_50,As_:3EC_50,Pb_, 2EC_50,As_:4EC_50,Pb_ and EC_50,As_:5EC_50,Pb_. At the same time, the equal concentration ratio of 1:10 (Rf) was selected as a reference based on the occurrence concentrations of As and Pb in solution to predict their interaction relationship.

### 2.3. Experimental Design

**Figure 2:**
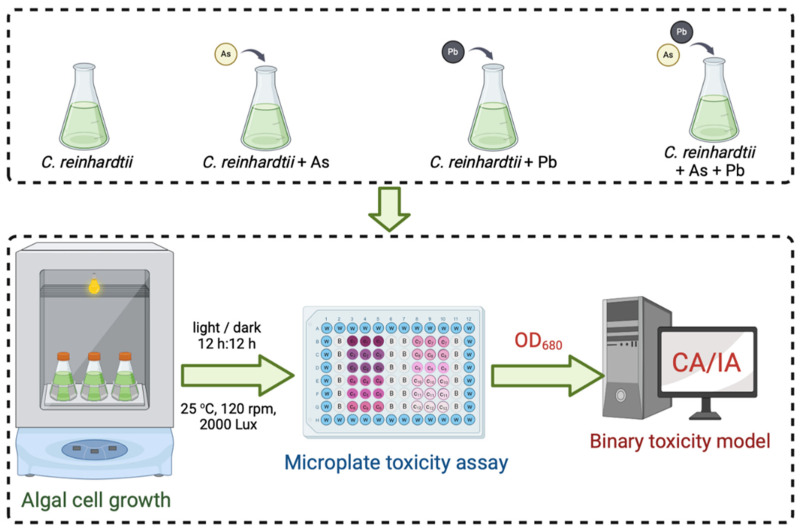
Experimental flow chart of As and Pb toxic effect model.

The experiment was setup with single and combined As(V) and Pb(II) treatments, and the growth inhibition effect of *C. reinhardtii* was tested in the concentration range of 1~98%. The experiment selected a 50 mL conical flask, added 20 mL TAP liquid culture medium, sterilized and cooled to room temperature for use. The activated *C. reinhardtii* in the logarithmic growth phase was inoculated into the culture medium, and 12 ion solution concentrations calculated according to Equation (2) were added (the treatment without heavy metal ion solution was used as a blank control), and 3 replicates were set for each treatment. Then a 96-well microplate (For the addition of microporous plates, see Figure S2) was used as a measurement carrier for toxicity testing. The half-maximum effect concentrations of single and combined As(V) and Pb(II) toxicity to *C. reinhardtii* were obtained to construct a toxic effect model, and on this basis, the interaction relationship between As and Pb was predicted when the As to Pb concentration ratio was 1:10. The experimental process is shown in Figure 2. The OD_680_ of each treatment was measured, and the growth inhibition rate E of the target pollutant on *C. reinhardtii* at the corresponding time point was calculated based on this. The calculation Equation is as follows:

(3) $$E=\frac{I_{0}-I_{i}}{I_{0}}\times 100\%$$

where $I_{0}$ is the average OD_680_ of the blank control, $I_{i}$ is the average OD_680_ of three times for each concentration gradient.

### 2.4. Concentration-Effect Curve and Best Curve Fitting

For experimental data with S-shaped concentration-effect curves, nonlinear fitting was performed on R, and OCIs with 95% confidence corresponding to CRC were calculated [22]. Since the optimization principle of chemometrics is that the number of samples used in an optimization model should be at least 5 times the parameters of the model, the 12 measured data points should be selected to use the 2-parameter Logit and Weibull nonlinear simulation CRC model [23].

Logit:   E = 1/(1 + exp(−α − β × log10(c)))(4)

Weibull:   E = 1 − exp(−exp(α + β × log10(c)))(5)

where c is the concentration of the pollutant; α and β are the location and slope parameters of Weibull and Logit; E is the effect, that is, the growth inhibition rate of the pollutant on green algae (0 ≤ E ≤ 1).

Given that the linear model has good estimation ability but not good prediction ability, a linear model is proposed, considering a set of n concentration response data points and a model function with m parameters. In this study, the experimental concentration (c) and growth inhibition rate (E) data were fitted to the nonlinear function using nonlinear least squares regression, and the goodness of fit of the model was evaluated by the following statistical data: adjusted fitting correlation coefficient (*R*^2^) and root mean square error (*RMSE*).

Best Curve Fitting, also known as all-subsets regression, is an optimization method that combines variable selection with multiple regression. The function with the larger value of the fitting correlation coefficient (*R*^2^) and the smaller value of the root mean square error (RMSE) is the best fitting function selected [23]. *R*^2^ and *RMSE* are shown in Equations (6) and (7).

(6) $$R^{2}=1-\frac{\left(n-1\right)\sum {\left(y_{i}-{\hat{y}}_{i}\right)}^{2}}{\left(n-m\right)\sum {\left(y_{i}-\bar{y}\right)}^{2}}$$

(7) $$RMSE=\sqrt{\frac{\sum_{i=1}^{n}{\left(y_{i}-{\hat{y}}_{i}\right)}^{2}}{n}}$$

where *n* is the number of observations; *m* is the number of parameters for the fitted model; $y_{i}$ is the response value; ${\hat{y}}_{i}$ is the fitted value; $\bar{y}$ is the average of the response values.

Then, 12 gradient concentrations of each ray were calculated according to Equations (1) and (2), and the toxicity corresponding to each concentration point was determined according to the above method. Five mixture concentration-response curves (m-CRCs) were established for each binary mixture.

### 2.5. Concentration Addition (CA) and Independent Action (IA) Models

To analyze the toxic interactions of mixtures more objectively and accurately, this experiment simultaneously used the CA model (Equation (8)) and the IA model (Equation (9)) to qualitatively evaluate the toxicity of the mixture. These models and their application follow established principles in aquatic toxicology [24,25,26]. If the upper and lower limits of the experimental point and its 95% OCI are between CA or IA, the mixture is additive; if the experimental point and its 95% OCI fall above CA or IA, the mixture is antagonistic; if the experimental point and its 95% OCI fall below CA or IA, the mixture is synergistic.

(8) $$\mathrm{CA}\;\mathrm{model}:\;\;\;{{EC}_{x,mix}=\left(\sum_{i=1}^{n}\frac{p_{i}}{{EC}_{x,i}}\right)}^{-1}$$

(9) $$\mathrm{IA}\;\mathrm{model}:\;\;\;x\%=1-\prod_{i=1}^{n}\left(1-f_{i}(p_{i}({EC}_{x,mix}))\right)$$

where ${EC}_{x,mix}$: The effect concentration corresponding to the *x%* effect of the mixture; ${EC}_{x,i}$: The effect concentration corresponding to the *x%* effect of the ith component when it exists alone; $p_{i}$: The concentration ratio of the component *i*; $f_{i}$: The best fitting function of the concentration-effect curve (CRC) of the component *i*.

### 2.6. Cell Morphology Analysis

In the experiment, we selected *C. reinhardtii* under the concentration ratio of As (200 μg/L) and Pb (2000 μg/L) of 1:10 and observed it under SEM and TEM.

#### 2.6.1. SEM Observation

After 96 h of being kept, 4 mL of the sample was spun to separate the parts using a centrifuge. The supernatant was discarded, and the algal cells were fixed with 2.5% glutaraldehyde solution for 30 min. Then, the sample was spun again at 8000 times per minute for 3 min to obtain a small pile of cells. A part of these was mixed with 5 mL of 50% alcohol. This was repeated with 75%, 85%, 95%, and 100% (*v*/*v*) alcohol. The dried sample was coated with gold to make it conduct electricity and then observed using a Supra 55 Scanning Electron Microscope (SEM, Zeiss, Germany).

#### 2.6.2. TEM Observation

The samples were colored using the double-staining method. Samples were fixed with 2.5% glutaraldehyde solution and 1% osmium acid solution at 4 and 20 °C for 2 h. Phosphate buffer (0.1 M, pH = 7.4) was used to wash after each fixing. Then the samples were dehydrated sequentially in an ethanol series (30%, 50%, 70%, 90%, 100%) for 15 min at each step. The samples were put into resin in an oven at 37 °C. Very thin slices of the biological material with a thickness of 60–100 nm was made using a Leica EM UC7 cutter and then put into a copper net. A TEM (Tecnai G2 F20S-TWIN, Thermo Fisher Scientific, Eindhoven, The Netherlands + AZtec 6.2 X-Max 80T) with a 200-keV Schottky field emission gun was used to study the tiny parts of *C. reinhardtii* cells.

### 2.7. Statistical Analysis

This paper comprehensively evaluates the toxic effects of mixtures based on the toxic effect model developed in the R platform (www.r-project.org, accessed on 3 April 2024) [27].

## 3. Results

### 3.1. Toxic Effects of Single Arsenic and Lead Treatments on C. reinhardtii

**Figure 3:**
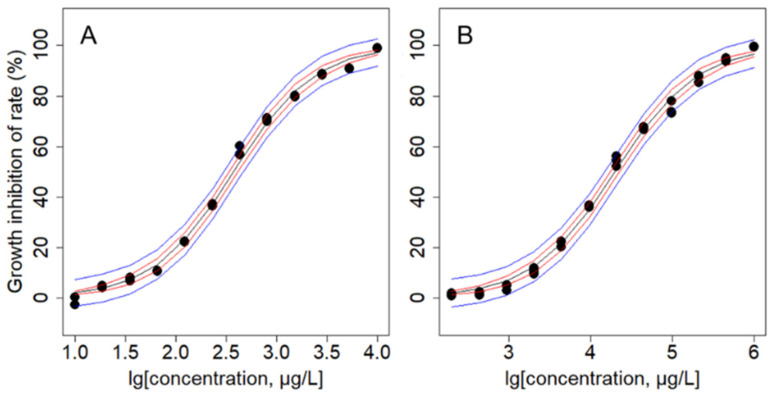
Concentration response curves of As (**A**) and Pb (**B**) to the cytotoxicity of *C. reinhardtii*. Dispersed solid point: experimental point; Black line: fitted CRC line; Red line: 95% FCI; The blue line: Lg stands for log base 10.

*C. reinhardtii* after 96 h incubation in TAP medium containing single arsenic and lead ions, respectively. the concentration-effect relationship was obtained (Figure 3) and the results of nonlinear Logit or Weibull function fitting were compared (Table 1).

The results in Table 1 show that the Logit function can better fit the concentration-effect relationship of arsenic and lead metal ions on *C. reinhardtii* at 96 h, with R^2^ greater than 0.996 and RMSE less than 0.025. The normal QQ plot of the residual (Figure S3) shows that the lower and upper ends of all As and Pb concentrations are symmetrically tailed. Different metal ions have different toxicities to *C. reinhardtii*. Taking EC_50_ as the toxicity index, arsenic is more toxic than lead at 96 h.

Figure 3 illustrates that the growth inhibition rate of the algae increases progressively with the elevated concentrations of single As and Pb. Both single arsenic and lead exerted inhibitory toxic effects on *C. reinhardtii*, and the curve is typically sigmoidal, with the steepest portion in the middle.

### 3.2. Combined Toxic Effects of Arsenic and Lead Binary Mixture on C. reinhardtii

The Direct equipartition ray design (EquRay) was utilized to devise a series of As-Pb binary mixtures with varying concentration ratios, as well as to predict the interactions at a fixed equimolar environmental concentration ratio (Rf) of As to Pb (1:10). The specific compositional components and concentration ratios of the binary mixtures are presented in Table 2.

The toxicological interaction relationships between As and Pb in the mixtures were qualitatively assessed using the CA and IA models. The concentration regions of each CEC can be broadly categorized into low concentration (<1 × 10^3^ μg/L), medium concentration (1 × 10^3^–2 × 10^4^ μg/L), and high concentration (>2 × 10^4^ μg/L) ranges.

Table 2 lists six CRC models, fitting regression parameters, statistics and EC_50_ values for equipartition ray fitting of As and Pb binary mixtures. It can be seen from Table 2 that six groups of different As and Pb mixture concentration ratios show different toxicity in the concentration-effect relationship within 96 h, and the toxicity increases gradually with the increase in As ions concentration, and can be well fitted by Logit function (R > 0.95). The normal QQ diagram (Figure S4) fitted with statistically significant residuals showed a symmetrical trailing distribution at the lower and upper ends of all the concentration ratios of As and Pb mixtures. Five concentration ratios (R1, R2, R3, R4 and R5) and environmental concentration ratio R_f_ (As:Pb = 1:10) composed of different combinations of As and Pb were selected, and six mixed systems with different ratios of As(V) and Pb(II) were selected to compare the toxicity of As and Pb. The results showed that R1 < R2 < R3 < R4 < R5 < R_f_.

**Figure 4:**
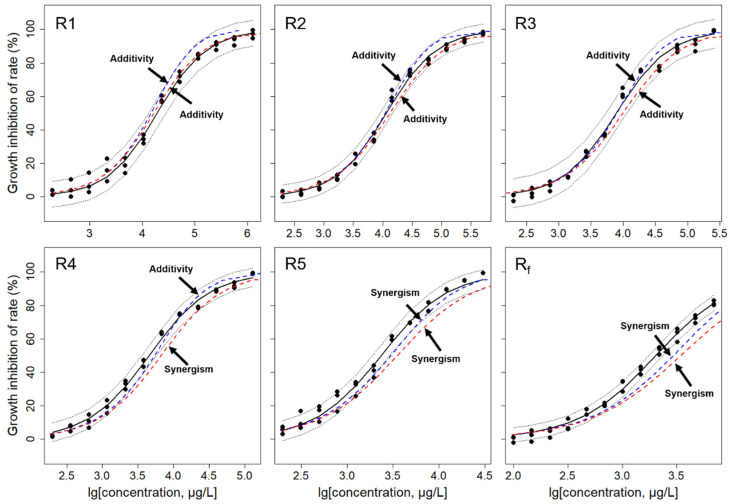
Evaluation of the interaction of each mixing ray for the As/Pb binary mixing system. Black scattered dots (●): experimental points; black solid line (—): fitted line; red dashed line (- - -): CA prediction line; blue dashed line (**- - -**): IA prediction line; black dotted line (- - -): 95% (confidence interval).

In order to describe the variation law of the concentration ratio of As and Pb mixtures more intuitively, we used 6 different concentration ratios. Figure 4 clearly demonstrates that these 6 groups of mixtures all show the characteristics of dependence on concentration ratio. Their toxic effects are the S-type, and the toxicity change trend is basically the same as that of single As/Pb. As depicted in Figure 4, in the binary mixed systems with different concentration ratios formed by As and Pb mixtures, as the concentration ratio of As and Pb mixtures increases, the toxicity intensity in the mixed system gradually increases with the increase in concentration, indicating that there is an interaction between the toxic effects of the As and Pb.

**Figure 5:**
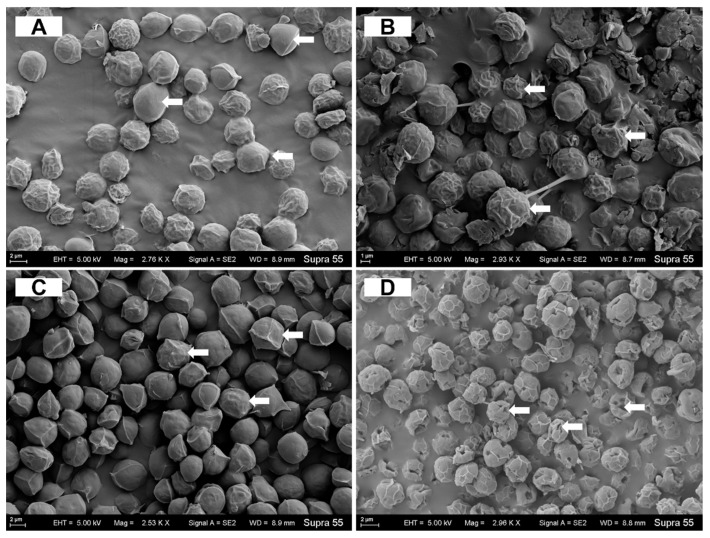
SEM images of *C. reinhardtii* cells without As/Pb (**A**), treated with 200 μg/L As (**B**), 2000 μg/L Pb (**C**) and 200 μg/L As + 2000 μg/L Pb (**D**). The arrows showed the morphological changes of the cells.

The SEM images revealed that at Pb (2000 μg/L) concentration, the cell surface morphology remained unaltered, and no depressions or breaks were observed when compared to the control group (Figure 5A,C). Conversely, at As (200 μg/L) concentration, the cell surface exhibited clear folds and depressions (Figure 5B). At the As (200 μg/L) + Pb (2000 μg/L) concentration, a significant increase in fold formation was observed, which subsequently aggregated and then collapsed inwards, resulting in the inward contraction of the cell structure to form depressions as well as cell breakage (Figure 5D).

**Figure 6:**
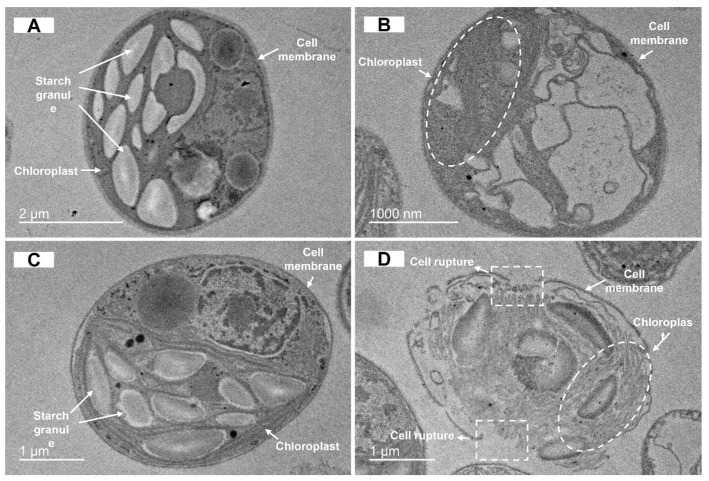
TEM images of C. reinhardtii cells without As/Pb (**A**), treated with 200 μg/L As (**B**), 2000 μg/L Pb (**C**) and 200 μg/L As + 2000 μg/L Pb (**D**).

The TEM results demonstrated that the cell structure exhibited no significant abnormalities or damage at Pb (2000 μg/L) concentrations compared with the control group and obvious starch granules could be observed (Figure 6A,C). However, at As (200 μg/L) concentrations, the cell morphology showed evident deformations. The cell wall exhibited a concave inward deviation, and the intracellular content became obscured (Figure 6B). In the concentration of As (200 μg/L) + Pb (2000 μg/L), the cell structure was more severely damaged, and the basic morphology was almost completely lost. The cell wall and cell membrane were predominantly ruptured, a significant quantity of content was discharged, and the chloroplast structure was also evidently damaged (Figure 6D).

## 4. Discussion

The maintenance of normal cellular structures is essential for organisms to sustain life processes, as compromised structures can disrupt metabolic activities and ultimately result in cell death [28]. Heavy metal and metalloid pollution typically impairs cellular structures in aquatic organisms, thereby affecting their metabolic functions and causing cell mortality [4]. The toxic effects on algal cells vary significantly between single heavy metal treatments and binary mixtures of different concentrations [8,15]. Therefore, by determining the EC_50_, which represents the growth inhibition rate of *C. reinhardtii* cells under 96 h exposure to As, Pb, and their mixtures of varying concentration ratios, combined with the actual environmental ratio of As and Pb mixtures, and selecting a representative concentration ratio of As:Pb = 1:10 for toxicity effect and model prediction analysis, a comprehensive assessment can be achieved.

In the case of single arsenic and lead treatments, both heavy metals exert inhibitory effects on *C. reinhardtii*, albeit with differing degrees of toxicity. After a 96 h exposure, the inhibition rates of both metals follow a classical sigmoidal curve, with As showing a greater inhibitory effect on algal growth than Pb. The median effect concentration (EC_50_) for As is two orders of magnitude lower than that for Pb. Using EC_50_ as a toxicity indicator, a significant difference in the lethal toxicity of As (EC_50_ = 374.87 μg/L) and Pb (EC_50_ = 19,988.75 μg/L) to *C. reinhardtii* is observed, which may be attributed to the fundamentally different inherent properties of the metalloid and metal and the distinct response mechanisms of the algae [29].

Since the introduction of Bliss’ concept of independent joint action and Loewe’s additivity [27,28], the Concentration Addition (CA) and Independent Action (IA) models have been widely applied in the toxicity assessment and prediction of chemical mixtures [12,29]. In the As/Pb mixture system, the six rays from R1 to Rf exhibit varying degrees of additivity and synergy (see Figure 4). Specifically, all concentrations of CA and IA lines for R1 to R3 fall within the 95% Observation-based Confidence Intervals (OCIs), indicating that the combined toxicity interactions on *C. reinhardtii* are additive, meaning the components act independently without significant synergy or antagonism at these ratios. However, the remaining three rays show different degrees of synergy. The CA and IA prediction lines for R4 and R5 exhibit additive effects at low concentrations and synergy at medium to high concentrations. Furthermore, as the concentration ratio of As and Pb mixtures increases, the toxicity interactions within the mixture system become more significantly synergistic at medium to high concentration ranges, demonstrating a typical concentration ratio-dependent relationship. The range of concentrations exhibiting synergy is broader, which may be related to the increasing concentration of As(V) and the potential influence of Pb on the toxicity of As. The synergistic effect of the As and Pb mixture at Rf (As:Pb = 1:10) is strongest when the effect is between 40% and 60%, a concentration ratio that closely aligns with environmental concentrations of As and Pb, thus possessing a high degree of environmental relevance and practical significance. These findings also indicate that the toxic interaction of As and Pb mixtures is significantly related to the increasing concentration of As and its proportion within the mixed heavy metals.

Electron microscopy can be used to directly observe the morphology change in *C. reinhardtii* cells to heavy metals. In the single system (As (200 μg/L) and Pb (2000 μg/L)), the toxicity of heavy metals to *C. reinhardtii* cells was found to be minimal. As shown in the results of TEM and SEM analyses, there was negligible change in cell morphology. This finding suggests that the *C. reinhardtii* cells could tolerate heavy metal stress within this system [30]. In addition, the significant increase in starch granules in the cells suggests that cells could synthesize large amounts of starch for binding and detoxification of arsenic and lead in both extracellular and intracellular compartments, in order to resist their toxicity [21]. However, a significant number of cell surface breakages and depressions were observed under TEM and SEM at As (200 μg/L) + Pb (2000 μg/L) concentrations, indicating that these resistance mechanisms in the joint system are not capable of enabling the cells to maintain normal life activities. The combined toxicity of arsenic and lead resulted in a more pronounced membrane structural impairment in *C. reinhardtii* cells (compared with the single system) due to a combination of arsenic-induced generation of ROS and lead-induced disruption of phospholipid structure [15].

## 5. Conclusions

This study systematically examined the individual and combined toxic effects of arsenic (As) and lead (Pb) on *C. reinhardtii*, demonstrating that As exhibits notably higher toxicity than Pb, with the combined toxicity influenced by concentration ratios. The interaction between these metals shifted from additive to synergistic with increasing As concentration, which we propose is likely due to the enhancing effect of Pb on As toxicity at higher As ratios. The concentration-response models developed and validated in this study offer a reliable predictive framework for assessing the combined toxicity of heavy metal mixtures, providing crucial insights to inform environmental risk assessment and pollution management strategies in aquatic ecosystems.

## Figures and Tables

**Table 1:** Function fitting results and statistics on concentration effect relationship.

Metal Ions	Fitting Function	Model Parameter				EC50 (μg/L)	NOEC (μg/L)	LOEC (μg/L)
		α	β	R^2^	RMSE			
As(V)	Logit	6.4033	2.4878	0.9963	0.0239	374.87	10.0000	18.7381
Pb(II)	Logit	8.5098	1.9786	0.9962	0.0243	19,988.75	433.8104	940.9575

NOEC: no observed effect concentration, LOEC: lowest observed effect concentration.

**Table 2:** Fitting parameters and interaction types of As-Pb mixture system.

Mixture Ray	Pas (%)	PPb (%)	Fitting Function	α	β	R^2^	RMSE	EC50 (μg/L)	CA	IA
R1	0.37	99.63	Logit	3.6333	2.1155	0.9931	0.03407	19,166.57	Additivity	Additivity
R2	0.93	99.07	Logit	4.5153	2.327	0.9964	0.02392	11,471.69	Additivity	Additivity
R3	1.84	98.16	Logit	5.0789	2.4099	0.9909	0.03804	7807.84	Additivity	Additivity
R4	3.61	96.39	Logit	5.1791	2.1769	0.9943	0.0278	4177.71	Synergism	Additivity
R5	8.57	91.43	Logit	7.2075	2.7393	0.9949	0.02584	2338.19	Synergism	Synergism
R_f_	9.09	90.91	Logit	7.4461	2.7688	0.9962	0.0187	2045.35	Synergism	Synergism

## Data Availability

Data is contained within the article or Supplementary Materials.

## Supplementary Materials

The following supporting information can be downloaded at: https://www.mdpi.com/article/10.3390/biology14101395/s1. Figure S1: OD values of *C. reinhardtii* cells at various time point (A); and at different wavelength (B); Figure S2: Schematic diagram of *C. reinhardtii* microplate loading. (W: deionized water; B: blank control (algae only); Ci: treatment group, i = 1,2…,12); Figure S3: The normal Q-Q plot of residuals for the curve fitting of As and Pb ionic liquids on *C. reinhardtii*; Figure S4: The normal Q-Q plot of residuals for the curve fitting of the different As and Pb ionic liquids concentration ratios on *C. reinhardtii*; Table S1: Chemical composition of TAP medium.
