# Supplementary material for: Model Construction and Prediction of Combined Toxicity of Arsenic(V) and Lead(II) on Chlamydomonas reinhardtii
Source: Biology (Basel). 2025 Oct 11;14(10):1395. doi: 10.3390/biology14101395 (PMC12561832; doi:10.3390/biology14101395)
Supplement: Supplementary file 1 [file biology-14-01395-s001.zip › biology-3899250-supplementary.pdf]

Supplementary Materials

Supplementary Table S1. Chemical composition of TAP medium

Reagent name	Reagent ingredients	Final concentration
Tris base	$H_2NC(CH_2OH)_3$	2.42 g/L
TAP salts	NH_4Cl	188.00 mg/L
	$MgSO_4$	50.00 mg/L
	$CaCl_2$•H_2O	25.00 mg/L
Phosphate solution	K_2HPO_4•$3H_2O$	1.40 mg/L
	KH_2PO_4	0.54 mg/L
Trace elements	$ZnSO_4$•$7H_2O$	22.00 mg/L
	H_3BO_3	11.04 mg/L
	$MnCl_2$•$4H_2O$	5.06 mg/L
	$CoCl_2$•$6H_2O$	1.61 mg/L
	$CuSO_4$•$5H_2O$	1.57 mg/L
	$(NH_4)_6Mo_7O_{24}$•$4H_2O$	1.10 mg/L
	$FeSO_4$•$7H_2O$	4.99 mg/L
	Na_2EDTA•$2H_2O$	50.00 mg/L
Acetic acid	CH_3COOH	1.00 mL/L

Determination of culture time: In order to monitor the various growth phases of *C. reinhardtii* cells during the actual culture process, 1 mL of *C. reinhardtii* cells in the logarithmic growth phase was added to 100 mL of TAP culture medium (P: 315 μg/L) before the experiment. Samples were taken every 12 hours for 120 hours to draw the growth curve of *C. reinhardtii* cells. In a sterile operating table, 200 μL of algae solution was added to the 36 microwells around the 96-well microplate. At the next corresponding time point, 200 μL of cultured *C. reinhardtii* cells were aspirated and the absorbance at a wavelength of 680 nm was measured using an ELISA reader. The results are shown in Figure S1A. The growth of *C. reinhardtii* cells in a conical flask conforms to the logistic growth curve and reaches a steady growth phase at 96 hours. The duration of the *C. reinhardtii* culture experiment was set to 96 hours.

Selection of measurement wavelength: 200 μL of *C. reinhardtii* cells in the logarithmic phase were aspirated into a 96-well microplate, and the optical density (OD) of *C. reinhardtii* cells was measured at a wavelength of 400-900 nm and a step length of 1 nm. The results are shown in Figure S1B. As can be seen from the figure, the absorption peaks exist at wavelengths of 620, 680, 735, and 875 nm, among which the absorption peak at 680 nm is the sharpest and has less spectral interference. Therefore, a wavelength of 680 nm was selected to measure the OD value of *C. reinhardtii* cells.

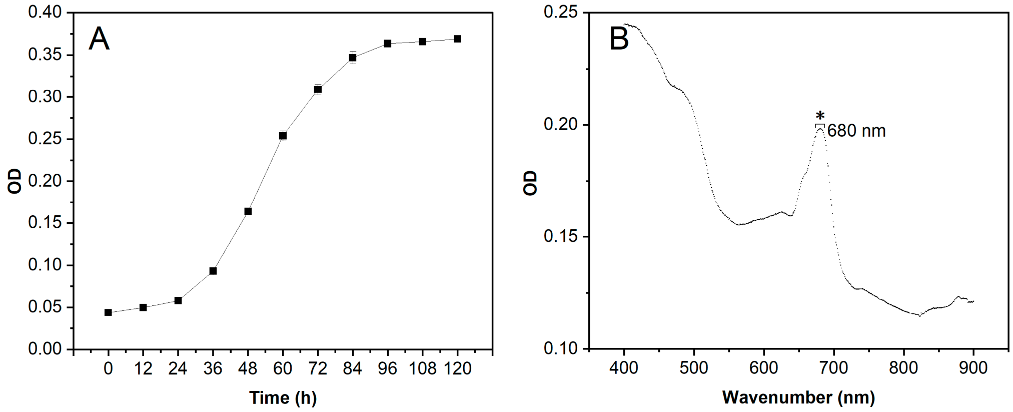

Supplementary Figure S1. OD values of *C. reinhardtii* cells at various time point (A); and at different wavelength (B).

A 96-well microplate was used as a measurement carrier for toxicity testing. The schematic diagram of the microplate loading is shown below. The steps for loading the microplate are as follows: 200 μL of deionized water is added to each well around the microplate to prevent marginal effects. Algae solution is added to the 24 wells in the 2nd, 6th, 7th and 11th columns of the remaining 60 wells. 200 μL of culture solution treated with 12 different concentrations of pollutants is added to the 6 wells in the 3rd column and the 6 wells in the 8th column (the corresponding concentrations are prepared with TAP liquid culture medium according to the determined dilution factor). The 4th and 5th columns are parallel experiments of the 3rd column, and the 9th and 10th columns are parallel experiments of the 8th column. The OD_{680} of each treatment is measured.

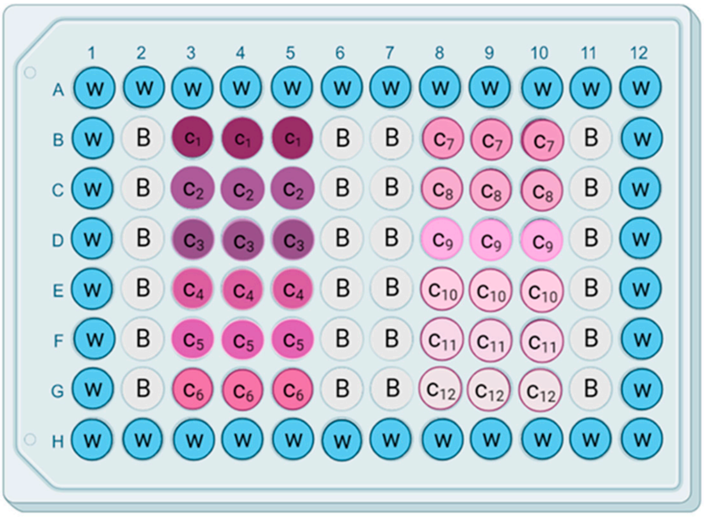

Supplementary Figure S2. Schematic diagram of *C. reinhardtii* microplate loading. (W: deionized water; B: blank control (algae only); Ci: treatment group, i=1,2…,12).

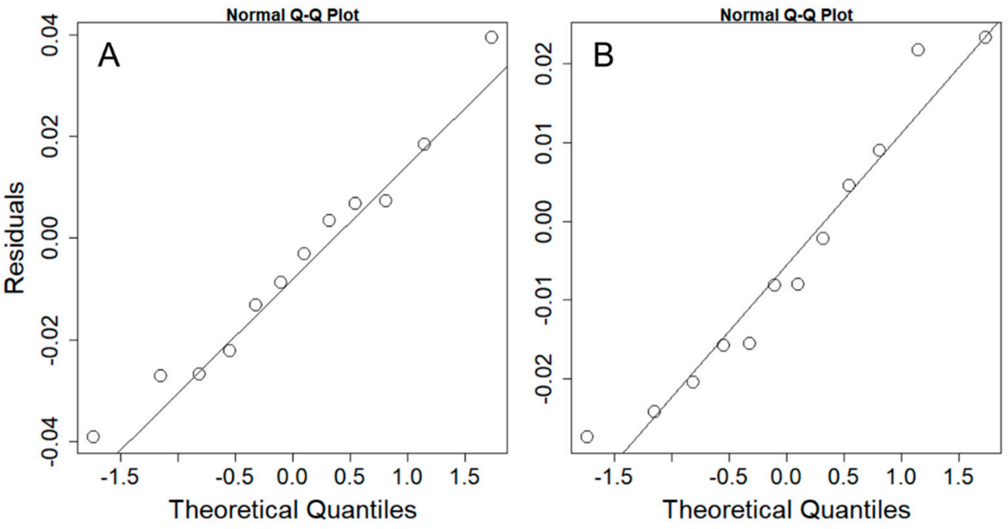

Supplementary Figure S3. The normal Q-Q plot of residuals for the curve fitting of As and Pb ionic liquids on *C. reinhardtii.*

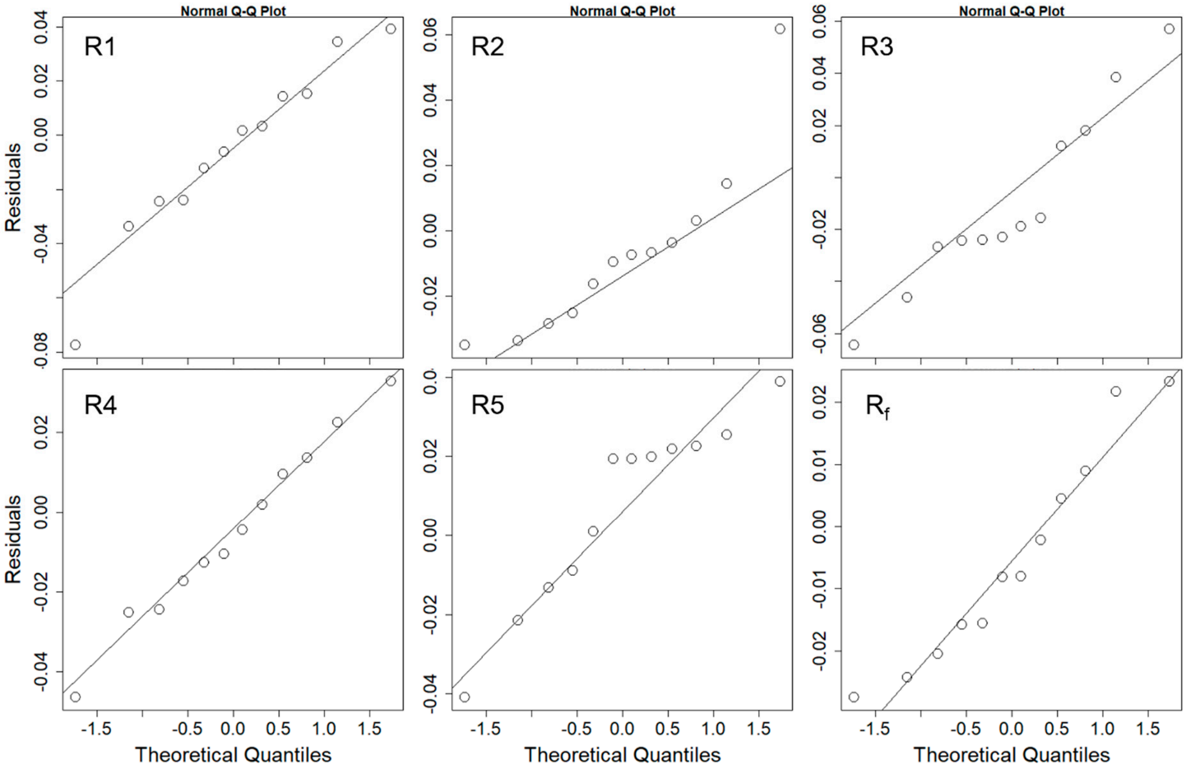

Supplementary Figure S4. The normal Q-Q plot of residuals for the curve fitting of the different As and Pb ionic liquids concentration ratios on *C. reinhardtii.*
